# Antidepressant activity: contribution of brain microdialysis in knock-out mice to the understanding of BDNF/5-HT transporter/5-HT autoreceptor interactions

**DOI:** 10.3389/fphar.2013.00098

**Published:** 2013-08-08

**Authors:** Alain M. Gardier

**Affiliations:** EA 3544 “Pharmacologie des troubles anxio-dépressifs et Neurogenèse”, Faculté de Pharmacie, Université Paris-SudChatenay-Malabry, France

**Keywords:** knock-out mice, antidepressants, autoreceptors, serotonin, BDNF, microdialysis

## Abstract

Why antidepressants vary in terms of efficacy is currently unclear. Despite the leadership of selective serotonin reuptake inhibitors (SSRIs) in the treatment of depression, the precise neurobiological mechanisms involved in their therapeutic action are poorly understood. A better knowledge of molecular interactions between monoaminergic system, pre- and post-synaptic partners, brain neuronal circuits and regions involved may help to overcome limitations of current treatments and identify new therapeutic targets. Intracerebral *in vivo* microdialysis (ICM) already provided important information about the brain mechanism of action of antidepressants first in anesthetized rats in the early 1990s, and since then in conscious wild-type or knock-out mice. The principle of ICM is based on the balance between release of neurotransmitters (e.g., monoamines) and reuptake by selective transporters [e.g., serotonin transporter for serotonin 5-hydroxytryptamine (5-HT)]. Complementary to electrophysiology, this technique reflects pre-synaptic monoamines release and intrasynaptic events corresponding to ≈80% of whole brain tissue content. The inhibitory role of serotonergic autoreceptors infers that they limit somatodendritic and nerve terminal 5-HT release. It has been proposed that activation of 5-HT_1A_ and 5-HT_1B_ receptor sub-types limits the antidepressant-like activity of SSRIs. This hypothesis is based partially on results obtained in ICM experiments performed in naïve, non-stressed rodents. The present review will first remind the principle and methodology of ICM performed in mice. The crucial need of developing animal models that display anxiety and depression-like behaviors, neurochemical and brain morphological phenotypes reminiscent of these mood disorders in humans, will be underlined. Recently developed genetic mouse models have been generated to independently manipulate 5-HT_1A_ auto and heteroreceptors and ICM helped to clarify the role of the pre-synaptic component, i.e., by measuring extracellular levels of neurotransmitters in serotonergic nerve terminal regions and raphe nuclei. Finally, we will summarize main advantages of using ICM in mice through recent examples obtained in knock-outs (drug infusion through the ICM probe allows the search of a correlation between changes in extracellular neurotransmitter levels and antidepressant-like activity) or alternatives (infusion of a small-interfering RNA suppressing receptor functions in the mouse brain). We will also focus this review on post-synaptic components such as brain-derived neurotrophic factor in adult hippocampus that plays a crucial role in the neurogenic and anxiolytic/antidepressant-like activity of chronic SSRI treatment. Limitations of ICM will also be considered.

## INTRODUCTION

Most of the antidepressants such as selective serotonin reuptake inhibitors (SSRIs) act as indirect agonists of monoamine receptors. While SSRI drugs produce relatively rapid blockade of serotonin [5-hydroxytryptamine (5-HT)] transporters (SERTs) *in vitro*, the onset of clinical benefits usually takes several (4–6) weeks to occur (Blier et al., 1987). This gap in timing between SSRI near-immediate effect on neurotransmitter systems and the slow symptomatic recovery is a paradox that has not been completely solved yet. At pre-synaptic level, SSRI-induced blockade of SERT results in a rapid suppression of the firing activity of 5-HT neurons in the brainstem (Blier, 2001): these results have been obtained by using an electrophysiological technique in anesthetized animals.

### MICRODIALYSIS: PRINCIPLE AND METHODOLOGY IN MICE

The principle of microdialysis technique is based on the balance between the release of neurotransmitters (e.g., 5-HT) and its reuptake (e.g., by SERT). Usually, male 3- to 4-month-old wild-type (WT) or mutant mice (25–30 g in body weight) are used for microdialysis experiments.

#### Conventional intracerebral in vivo microdialysis

Whole brain tissue measurements represent a mixture of the intracellular (≈20%) and extracellular (≈80%) content. To obtain a measurement more directly related to synaptic transmission, it is interesting to sample specifically the content of the extracellular space, which is the site of exchanges between neurons, glial cells, and blood vessels (Zetterström et al., 1983). It contains various monoamines, excitatory and inhibitory amino acids, neuropeptides and their metabolites as well as precursors of these neurotransmitters. In the mid-1980s, the development of very sensitive analytical techniques such as liquid chromatography and electrochemical detection (LC-ED) had made possible to perform *in vivo* microdialysis first in anesthetized rodents, then in awake, freely moving animals.

**FIGURE 1:**
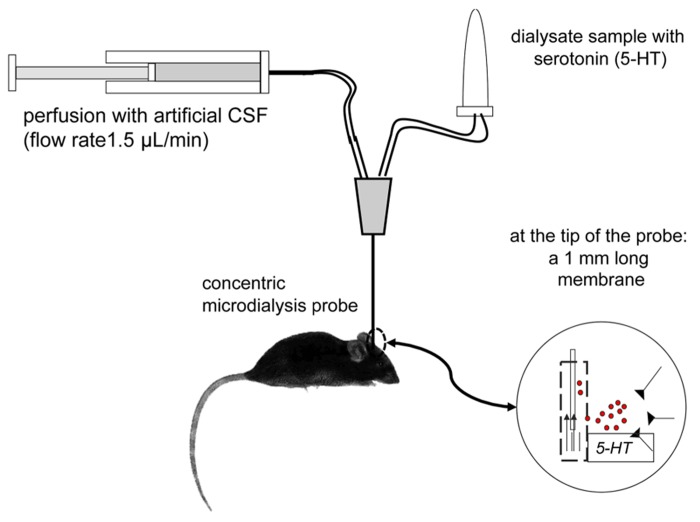
**Principle of intracerebral microdialysis in awake, freely moving mice**.

*In vivo* microdialysis technique, in anesthetized or awake animals, was developed by the group of Delgado et al. (1972) in monkeys and then improved in rats by the group of Ungerstedt (Zetterström et al., 1983) in the early 1980s. It is based on the law of passive diffusion of low molecular-weight compounds through a porous membrane from the compartment with the highest concentration of neurotransmitters (the synaptic extracellular space) to the less concentrated compartment (i.e., the dialysis probe perfused with a buffer solution at physiological pH that does not contain neurotransmitters; **Figure 1**). This technique, now currently applied in our laboratory in awake, freely moving WT control or knock-out (KO) adult mice, allows the collection of samples (named “dialysates”) every 10 or 20 min with a flow rate from 0.5 to 1.5 μl/min depending on the experimental protocol and the brain region studied. These samples contain, among other molecules, serotonin, its major metabolite (5-HIAA) and norepinephrine (NE), dopamine (DA), and their metabolites. These molecules are then quantified by using high-performance LC coupled to an amperometric detector (e.g., 1049A, Hewlett-Packard, Les Ulis, France). The limit of sensitivity for 5-HT is ~0.5 fmol/sample (signal-to-noise ratio = 2).

The concentrations of neurotransmitters reflect the physiological balance between the calcium-dependent neurotransmitter release and its reuptake by SERT located on the membrane of pre-synaptic neurons. A comprehensive study of intracerebral microdialysis has four phases: (1) surgical stereotaxic implantation of the probe under anesthesia, (2) the collection of dialysates (first to measure baseline value of extracellular neurotransmitter levels before and 2–3 h after drug treatment), (3) the collection of brains for the accurate verification of the implantation site of the microdialysis membrane, and (4) of chromatographic analysis of dialysate samples (see Malagié et al., 2001; Guiard et al., 2004 for details).

#### Drug administration by reverse microdialysis

A major advantage of the microdialysis technique is to infuse a drug locally into the brain to confirm central effects on dialysates first measured following a peripheral injection of the drug. Thus, drugs with a high molecular weight can be dissolved in artificial cerebrospinal fluid (aCSF) and administered locally, for example, into the ventral hippocampus *via* a silica catheter glued to the microdialysis probe (flow rate: 0.2 μl/min for 2 min), at the dose of 10–100 ng (Guiard et al., 2007; Deltheil et al., 2008). For each experiment, a control group must receive the appropriate vehicle.

#### Zero net flux method of quantitative* intracerebral microdialysis

The zero net flux method of quantitative microdialysis is used to quantify basal extracellular neurotransmitter concentrations and the extraction fraction (*E*_d_) of this neurotransmitter, which provides an index of the functional status of the neurotransmitter uptake *in vivo*. Usually, four samples are collected to determine basal hippocampal 5-HT levels (as in David et al., 2004 in NK1 receptor KO mice), before local perfusion of increasing concentrations of 5-HT (0, 5, 10, and 20 nM). The dialysate 5-HT concentrations (*C*_out_) obtained during perfusion of the various concentrations of 5-HT (*C*_in_) are used to construct a linear regression curve for each animal (Guiard et al., 2008). The net change in 5-HT (*C*_in_-*C*_out_) is plotted on the *y*-axis against *C*_in_ on the *x*-axis. Extracellular 5-HT levels ([5-HT]_ext_) and the extraction fraction of the probe (*E*_d_) are determined as described by Parsons et al. (1991). The concentration of 5-HT in the extracellular space is estimated from the concentration at which *C*_in_-*C*_out_ = 0 and corresponds to a point at which there is no net diffusion of 5-HT across the dialysis membrane. The extraction fraction (*E*_d_) is the slope of the linear regression curve and has been shown to provide an estimate of changes in transporter-mediated 5-HT uptake (Parsons et al., 1991; Gardier et al., 2003).

As an example of the relevance of the zero net flux method of quantitative microdialysis, we have recently shown the critical impact of a neuropeptide, brain-derived neurotrophic factor (BDNF) on serotonergic neurotransmission under basal conditions and following SSRI treatment. In a series of experiments, we examined the consequences of either a constitutive decrease (Guiard et al., 2008) or increase in brain BDNF protein levels (Benmansour et al., 2008; Deltheil et al., 2008, 2009) on hippocampal extracellular levels of 5-HT in conscious mice. The no net flux method allows unveiling differences in basal extracellular 5-HT levels in heterozygous BDNF^+^^/^^-^ mice (Guiard et al., 2008). Indeed, this neurotrophic factor is known to play a role in mood disorders and the mechanism of action of antidepressant drugs. However, the relationship between BDNF and serotonergic signaling is poorly understood. BDNF^+^^/^^-^ mice were used to investigate the influence of BDNF on the 5-HT system and the activity of SERT in the hippocampus. The zero net flux method revealed that these mutants have increased basal extracellular 5-HT levels in the hippocampus and decreased 5-HT reuptake capacity. These results are coherent with the lack of effect of paroxetine to increase hippocampal 5-HText levels in BDNF^+^^/^^-^ mice, while it produced robust effects in WT littermates. As expected, *in vitro* autoradiography and synaptosome techniques in BDNF^+^^/^^-^ mice revealed a significant decrease in [3H]citalopram-binding-site density in the CA3 subregion of the ventral hippocampus and a significant reduction in [3H]5-HT uptake in hippocampal synaptosomes. Taken together, these results provide evidence that constitutive reductions in BDNF modulate SERT function reuptake in the hippocampus.

#### Statistical analysis and expression of results of microdialysis experiments in KO mice

Usually, microdialysis data are reported as means ± SEM. For conventional microdialysis experiments, we used to perform statistical analyses on areas under the curve (AUC) values for the amount of 5-HT outflow collected during the 0–120 min post-treatment period. To compare different AUC values in each group of mice, a two-way ANOVA with genotype factor and treatment factor is performed. We used to present microdialysis data as histograms because statistical analysis on AUC values better reflects the pharmacological properties of a compound than the kinetics. We strongly believe that the interpretation of these data is more appropriate when performed on AUC values in dialysate 5-HT levels (Guilloux et al., 2011; Nguyen et al., 2013) as well as for DA levels (Maskos et al., 2005; Reperant et al., 2010) when changes induced by drugs are compared between WT versus KO mice.

Using intracerebral microdialysis in the hippocampus and cortex in mice, measuring statistically significant changes in dialysate 5-HT levels induced, for example, by a given drug between *t*_30_ min and *t*_45_ min offers little interest. We feel that these information make the message more difficult to interpret and do not fundamentally improve the study. These time courses are strongly dependent on the experimental conditions and consequently not reproducible between laboratories. By contrast, our experience reveals that comparable results from distinct laboratories can be obtained from the analysis of AUC values. The inclusion of the data showing the time course for the microdialysis is often superfluous. Microdialysis is a neurochemical technique, not sensitive enough to explore precisely (i.e., sample-by-sample) the time course of drug effects.

**FIGURE 2:**
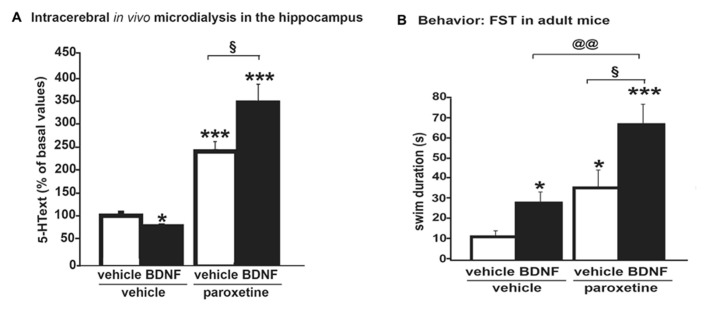
**(A)** Microdialysis data showing that an acute intra-hippocampal injection of BDNF (100 ng) potentiated the effects of the systemic administration of an SSRI, paroxetine (4 mg/kg; i.p.) on dialysate 5-HText in the hippocampus of freely moving wild-type mice. Results are expressed as AUC values (means ± SEM) calculated for the amount of 5-HText collected during the 0–120 min post-treatment period. **(B)** Antidepressant-like activity of paroxetine as measured on swimming behavior in the forced swim test (FST) was potentiated by BDNF. Thus, neurochemical changes correlated with behavioral data in this protocol, suggesting that a BDNF + SSRI combination may offer new alternatives to treat mood disorders (from Deltheil et al., 2009). *p < 0.05, ***p < 0.001 when compared to the vehicle-treated group; ^§^*p* < 0.05 when compared to the paroxetine/vehicle-treated group and paroxetine/BDNF-treated group; ^@@^*p* < 0.01 when compared to the BDNF/vehicle-treated group and BDNF/paroxetine-treated group (two-way ANOVA, Fisher’s PLSD *post hoc* test).

However, in some cases, it is interesting to show the time course analysis of the microdialysis data:

(1) when we need to express time course data in microdialysis experiments as concentrations (in fmol/sample, not as % changes) because the baseline dialysate levels of the neurotransmitter are statistically different between two groups of mice, i.e., in **Table 1** and **Figures 2** and **3** in Guiard et al. (2008): heterozygous BDNF^+^^/^^-^ mice had a higher basal 5-HText levels in the hippocampus compared to WT mice. See also in **Table 1** and Figure 6 in Guilloux et al. (2011), in which double 5-HT_1A_/1B^-^^/^^-^ mice display a higher basal 5-HText levels in the frontal cortex and dorsal raphe nucleus (DRN) compared to WT mice.

**Table 1:** Summary of the main advantages and some critical points of the intracerebral microdialysis technique in freely moving mice.

Main advantages of using microdialysis in WT and KO mice^*^	Some limitations of using microdialysis in WT and KO mice
– *In vivo* pre-synaptic test to study consequences of autoreceptor of transporter blockade on release and reuptake of neurotransmitters. – Direct access of exogenous molecules into the brain tissue, with minimal damage: an ideal approach to confirm brain effects observed following a systemic administration. Even more interesting when the drug does not cross easily the blood brain barrier such as molecules with a high molecular weight: neurotrophic factors, e.g., BDNF (Benmansour et al., 2008; Deltheil et al., 2009); substance P (Guiard et al., 2007) – ^*^To validate the KO animal model: – ^*^Possibility to implant two probes in the same mouse: a probe at the vicinity of cell bodies (e.g., raphe nuclei when studying the neuronal 5-HT system), and a probe at serotonergic nerve terminals (hippocampus, frontal cortex), thus evaluating a neural circuit – ^*^Possibility of measuring several neurotransmitters in the same dialysate sample of WT and KO mice (Nguyen et al., 2013). – ^*^The same of WT or KO mouse can be studied for two consecutive days, e.g., on day 1 following administration of the vehicle in the control group, and on day 2 following the novel pharmacological treatment – ^*^Chronic microdialysis: when using a guide cannula, it is possible to collect samples once a week for several weeks in the same WT or KO mouse (Popa et al., 2010) – When applied in awake, freely moving animals, functional consequences of SSRI-induced increases in extracellular neurotransmitter levels can be studied, e.g., correlation between changes in brain 5-HText and behavioral data (the swimming time in the FST, for example (Deltheil et al., 2009; Nguyen et al., 2013)	– Compared to electrophysiology, technique of reference ° Large outer diameter of microdialysis probe (0.2 mm) ° During microdialysis experiments, the samples are collected every 15–20 min (in the hippocampus and frontal cortex), every 10 min in raphe nuclei. This is due to the slow flow rate of the perfusion medium (≈ μl/min), which leads to a poor temporal resolution compared to electrophysiology (400 ms) – Time consuming: ° One experimenter, two mice, 1 day; 10–12 animals per group; delayed results (HPLC). Possible improvement with more sensitive analytical methods such as capillary electrophoresis coupled to a laser-induced fluorescence detection (Parrot et al., 2007; Denoroy et al., 2008), but it remains a very complex technique. ° 3–6 months to complete an experiment, i.e., to evaluate the effects of several doses of an agonist-antagonist compared to mice treated with the vehicle or in WT controls. Even longer when using Tg or KO mice (breeding, genotyping, selection of age, sex, and so on…). – Delicate animal handling, to avoid effects of stress, thus requiring an experienced experimenter to perform *in vivo* microdialysis in freely moving mice. – Absolute need to check the exact location of the probe, macroscopically on brain coronal sections at the end of the experiment. Especially in mice (Bert et al., 2004) – Poor prognostic value of *basal* extracellular concentrations of 5-HT, DA, and NA. – Extracellular concentrations of metabolites in dialysates (e.g., 5-HIAA, the main metabolite of 5-HT): ° *Under basal conditions*: it reflects intracellular metabolism of 5-HT (MAO A activity), and not its release or utilization (Wolf et al., 1985; Bel and Artigas, 1996) ° *Following pharmacological treatment: *it has little interest because dialysate 5-HIAA levels decrease, independently of the dose of the indirect 5-HT receptor agonist administered. These changes are not related to the neuronal activity (Malagié et al., 1995; Rocher et al., 1996).

* Some advantages of this technique are very interesting in KO mice knowing the difficulties to breed most of them.

**FIGURE 3:**
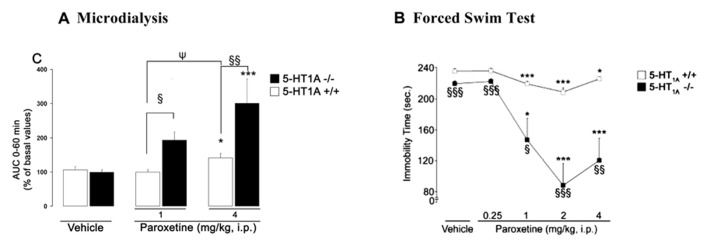
**(A)** Microdialysis data showing the effects of paroxetine on cortical 5-HText in 5-HT_1A_^+^^/^^+^ wild-type and 5-HT_1A_^-^^/^^-^ mice. Results are expressed as AUC values (means ± SEM) calculated for the amount of 5-HText collected during the 0–60 min post-treatment period. **(B)** Antidepressant-like effects of paroxetine on the immobility time in the mouse forced swimming test (FST) in 5-HT_1A_^+^^/^^+^ and 5-HT_1A_^-^^/^^-^ mice. FST and microdialysis experiments have been performed separately. Microdialysis and behavioral experiments were carried out by using the same experimental protocol. The duration time of the FST was 6 min, performed at the maximum effect of paroxetine on dialysate 5-HText, i.e., 30 min after its administration (from Guilloux et al., 2006). **(A)** *p < 0.05; ***p < 0.001 when compared to the appropriate vehicle-treated group; ^§^*p* < 0.05; ^§§^*p* < 0.01 when compared to 5-HT_1A_^+^^/^^+^ control mice; ^ψ^*p* < 0.05 when compared to the paroxetine 1 mg/kg-treated group (two-way ANOVA followed by a PLSD *post hoc* *t*-test). **(B)** *p < 0.05; ***p < 0.001 when compared to the appropriate control group; ^§^*p* < 0.05; ^§§^*p* < 0.01; ^§§§^*p* < 0.001 when compared to 5-HT_1A_^+^^/^^+^ mice. Statistical analysis was carried out using a two-way ANOVA followed by Fisher’s PLSD *post hoc* *t*-test.

(2) when it is sometimes important to collect some pharmacokinetic information about the short-term or long-lasting effect of a new drug in rodents. The AUC analysis of microdialysis data disregards information about differences in *C*_max_ and duration of the drug effects.
(3) when a gray line (**Figure 3** in Guilloux et al., 2006; **Figures 1** and **2** in Nguyen et al., 2013) indicates the duration time of the forced swim test (FST, i.e., 6 min), which was performed, in a separate group of animal, at the maximum effect of the antidepressant on cortical extracellular 5-HT levels in mice. It emphasizes that microdialysis and behavioral experiments were carried out by using the same experimental protocol.

## INTRACEREBRAL *IN VIVO* MICRODIALYSIS IN RODENTS

Another technique has provided complementary information about the mechanism of action of SSRIs: intracerebral *in vivo* microdialysis (ICM) performed in awake, freely moving animals (first in rats, now in mice). Information included in this chapter was drawn from our own experience in this field and relevant publications from other investigators.

### FIRST IN RATS

When it was first used in rat brain in the mid-1980s, this technique measured, for example, extracellular concentrations of monoamines such as serotonin (5-HText), which reflect pre-synaptic release of 5-HT and intrasynaptic events. With its coupling to very sensitive analytical techniques, it has provided much information regarding changes in the local pre-synaptic release of monoamines following acute drug administration. Thus, it has been possible to obtain two major arguments supporting the hypothesis that somatodendritic 5-HT_1A_ autoreceptors located in the raphe nuclei play an important role in the mechanism of action of SSRIs in rats (Gardier et al., 1996). At first, we have learned that a single administration of SSRIs at low doses comparable to those used therapeutically increased 5-HText in the vicinity of the cell body and the dendrites of serotoninergic neurones of the DRN (Malagié et al., 1995). This effect was more pronounced than that observed in regions rich in nerve endings (frontal cortex, ventral hippocampus; Malagié et al., 1996), probably due to a higher SERT density (Hrdina et al., 1990). Hence, the magnitude of the activation of the serotonergic neurotransmission depends on the brain area studied and the dose of the SSRIs administered to rats. This difference has been attributed to the activation of somatodendritic 5-HT_1A_ autoreceptors by endogenous 5-HT in the raphe nuclei, thereby limiting the corticofrontal effects of the antidepressant. Microdialysis technique demonstrated that despite SSRI-induced 5-HT reuptake inhibition also taking place at nerve terminals, there is a decrease in 5-HT release *via* activation of 5-HT_1A_ (somatodendritic) or 5-HT_1B_ (nerve terminal) autoreceptors (Rutter et al., 1995). Thus, depending on the terminal 5-HT brain area, only a small increase or no change at all in the synaptic availability of 5-HT occurs (Malagié et al., 1996; Romero et al., 1996). These microdialysis results obtained in rats have then been extended to measure SSRI-induced changes in DRN 5-HText in awake, freely moving KO mice (Bortolozzi et al., 2004; Guiard et al., 2004).

Next, we have learned from microdialysis performed in rats that SSRIs cause a larger increase in 5-HText at nerve endings following an acute treatment *versus* a chronic one. As the treatment is prolonged, a robust and time-dependent downregulation of SERT was observed (Pineyro et al., 1994; Benmansour et al., 2002), while 5-HT_1A_ autoreceptors gradually desensitize leading to a progressive recovery to normal of the firing rate of 5-HT neurons (Blier et al., 1986; Chaput et al., 1986; El Mansari et al., 2005). However, these molecular events seem to depend on 5-HT_1A_ autoreceptor internalization (Popa et al., 2010). Indeed, we studied the function of the 5-HT system in the raphe nuclei and hippocampus by using repeated *in vivo* microdialysis sessions in awake, freely moving mice. We assessed the degree of 5-HT_1A_ autoreceptor desensitization by using a local infusion of the 5-HT_1A_ receptor antagonist, WAY 100635, in the raphe *via* reverse microdialysis. We found that the anxiolytic-like effects of fluoxetine correlate in time and amplitude with 5-HT_1A_ autoreceptor desensitization, but neither with the basal extracellular levels of 5-HT in the raphe nuclei, nor in the hippocampus. These results suggests that the beneficial anxiolytic/antidepressant-like effects of chronic SSRI treatment depend on 5-HT_1A_ autoreceptor internalization, but do not require a sustained increase in extracellular 5-HT levels in a territory of 5-HT projection such as hippocampus. Several studies of patients with depression appear to confirm these experimental results, suggesting that co-administration of a 5-HT_1A_ autoreceptor antagonist (pindolol) and an SSRI accelerated the onset of the antidepressant effect (Portella et al., 2011). However, given the complex pharmacology of pindolol, new drug developments may help to discover either selective and silent 5-HT_1A_ receptor antagonists to be prescribed in combination with SSRIs, or dual action agents (SSRI + 5-HT_1A_ receptor antagonists; Artigas et al., 2006).

### NEXT IN WILD-TYPE AND KNOCK-OUT MICE

#### The use of pharmacological tools in mice

Changes in the amount of neurotransmitters (mainly monoamines such as 5-HT, NE, and DA) in synapses can be viewed as near-immediate effects of SSRI on brain neurotransmitter systems. *In vivo* brain microdialysis allows to measure basal extracellular levels of these neurotransmitters giving an idea of neurochemical events occurring at nerve terminals in brain regions of awake, freely moving rodents. In our laboratory, we extensively applied this technique in genetic and pharmacological studies aimed at investigating the relationship between neurotransmitters and brain regions, or between neurochemical changes and animal behaviors (see examples below). Among the main interests of microdialysis application is the infusion of drugs through the microdialysis probe (reverse dialysis) in conscious KO mice as well as in WT mice used as controls in these pharmacological experiments (e.g., intra-raphe perfusion of substance P in Guiard et al., 2007; BDNF in Deltheil et al., 2009).

As already mentioned, most prescribed serotonergic antidepressants show limited efficacy and delayed onset of action, partly due to the activation of somatodendritic 5-HT_1A_ autoreceptors by the excess extracellular 5-HT produced by SSRI in the raphe nuclei. A group of scientists in Spain recently addressed this problem using an original strategy. Bortolozzi et al. (2012) administered a small-interfering RNA (siRNA) to suppress acutely 5-HT_1A_ autoreceptor-mediated negative feedback mechanisms in the mouse brain. They developed a conjugated siRNA (C-1A-siRNA) by covalently binding siRNA targeting 5-HT_1A_ receptor mRNA with the SSRI sertraline in order to concentrate it in serotonin axons, rich in SERT sites. The intracerebroventricular (I.C.V.) infusion of C-1A-siRNA to mice resulted in its selective accumulation in serotonin neurons. This was associated with antidepressant-like effects in the forced swim and tail suspension tests, but did not affect anxiety-like behaviors in the elevated plus-maze. In addition, C-1A-siRNA administration markedly decreased 5-HT_1A_ autoreceptor expression and suppressed 8-OH-DPAT DPAT, i.p., SNRI [7-(dipropylamino)-5,6,7,8-tetrahydronaphthalen-1-ol]-induced hypothermia (a pre-synaptic 5-HT_1A_ receptor effect in mice) without affecting post-synaptic 5-HT_1A_ receptor expression in the hippocampus and prefrontal cortex. Moreover, I.C.V. C-1A-siRNA infusion augmented the increase in cortical dialysate 5-HT levels induced by fluoxetine to the level measured in 5-HT_1A_ receptor KO mice. Hence, C-1A-siRNA represents a new approach to treat mood disorders as monotherapy or in combination with SSRI.

To learn whether or not the *in vitro* affinity of SSRIs toward monoamine transporters can predict *in vivo* microdialysis data, we studied whether a single administration of a range of doses [1, 4, and 8 mg/kg, given intraperitoneally (i.p.)] of paroxetine, citalopram, or venlafaxine may simultaneously increase dialysate 5-HText and norepinephrine (NEext) by using *in vivo* microdialysis in the frontal cortex of awake, freely moving mice (David et al., 2003). We found that citalopram and paroxetine have the highest potency to increase cortical 5-HText and NEext, respectively. In addition, the rank of order of efficacy of these antidepressant drugs to increase cortical 5-HText *in vivo* in mice was as follows: venlafaxine > citalopram > paroxetine, while the efficacy to increase cortical NEext in mice of paroxetine and citalopram is similar, and greater than that of venlafaxine. Thus, the highest doses of the very selective SSRI citalopram and the very potent SSRI paroxetine were able to increase cortical NEext. Surprisingly, the serotonin-norepinephrine reuptake inhibitor (SNRI) venlafaxine increased cortical 5-HText to a greater extent rather than NEext in the range of doses studied in mice.

We recently confirmed these data with escitalopram, the *S*(+)-enantiomer of citalopram. To analyze the mechanisms by which SSRIs activate noradrenergic transmission in the brain, we compared the effects of escitalopram on both 5-HText and NEext in the frontal cortex of WT versus mutant mice lacking the 5-HT transporter (SERT^-^^/^^-^; Nguyen et al., 2013). In particular, the possibilities that escitalopram enhances NEext either by a direct mechanism involving the inhibition of the low- or high-affinity NE transporters or by an indirect mechanism promoted by 5-HText elevation were explored. The FST was used to investigate whether enhancing cortical 5-HText and/or NEext affected the antidepressant-like activity of escitalopram. As expected, a single systemic administration of escitalopram increased cortical 5-HText and NEext in WT mice. However, escitalopram failed to increase cortical 5-HText in SERT^-^^/^^-^ mice, whereas its neurochemical effects on NEext persisted in these mutants. In WT mice, these neurochemical changes induced by escitalopram were associated with increased swimming parameter in the FST. Finally, escitalopram, at relevant concentrations, failed to inhibit cortical NE and 5-HT uptake mediated by low-affinity monoamine transporters (i.e., organic cation transporters such as OCT1, 2, or 3). These experiments suggest that escitalopram enhances, although moderately, cortical NEext *in vivo* by a direct mechanism involving the inhibition of the high-affinity NE transporter (NET). Such *in vivo* effects of SSRIs could not be predicted by measuring the *in vitro* affinity of SSRIs toward SERT and NET in brain synaptosomes.

These results are not surprising. Indeed, experimental conditions (rat versus mice; whole brain versus cortical membranes; cell bodies versus nerve terminal regions; etc.) highly influence the values of binding parameters of ligands to neurotransmitter receptors or transporters measured *in vitro* (*B*_max_, *K*_D_, GTP-gammaS binding, etc.). The potency and selectivity of SSRIs as determined *in vitro* do not take into account noradrenergic projections and others, which obviously interfere *in vivo*, but not *in vitro*. Thus, function of monoamines transporters are much more complex than previously thought. *In vivo* experiments help to depict this complexity when it is possible to measure correlation between neurochemical parameters and behavior paradigms.

#### The use of mutated mice

The mouse genome can be specifically manipulated to produce the targeted deletion, replacement of genes, or down-/over-expression of related proteins in the brain (Sotnikova and Gainetdinov, 2007). This was first obtained in embryonic stem (ES) cells, but more recently, temporal and spatial controls of gene expression were possible in adult mice. In the field of anxiety and depression, preclinical studies such as those described above, have been mostly performed in healthy, “not depressed” animals. In the mid-1990s, genetically manipulated mice became available. It complicated the experimental protocol because it was necessary to include littermates as WT control mice. Great hopes were placed in mutant lines, some of them being considered as putative animal models of anxiety or depression. Several lines of transgenic (Tg) mice (carrying a human gene) or KO mice (i.e., homozygous mice lacking the two copies of a gene coding for a receptor or transporter of neurotransmitter or neuropeptide) were generated between 1994 and 1998. The first KO mice were generated by homologous recombination in the laboratory of S. Tonegawa at MIT (Silva et al., 1992).

The mouse is a model organism of choice in the field of neurosciences because (i) numerous genes have a human equivalent, (ii) many biological and biochemical functions of the mouse are similar to those of humans, and (iii) the genome mouse is easily manipulated by homologous recombination. This technique allowed the creation of animal-related patterns of human brain pathologies. The genetic background is a fundamental parameter for analyzing the phenotype of KO mice. Historically, the mutant mice were established using ES line 129/Sv. However, creating new lines of mutant mice on a genetic background C57BL/6 is now preferred, although there are limits on the use of this strain in some behavioral tests (see Gardier, 2009 for a review).

At that time, the procedure of ICM needed to be quickly adapted to perform experiments in an animal model having a smaller brain size than rats. Microdialysis experiments were first performed in tyrosine hydroxylase Tg mice by Nakahara et al. (1993). Then, it was applied to 5-HT_1B_ receptor KO mice (Saudou et al., 1994; Trillat et al., 1997), to DA transporter (DAT) KO mice (Gainetdinov et al., 1997), and so on. Of course, at the end of the experiments, the precise location of the microdialysis probe must be macroscopically verified according to the stereotaxic coordinates given by the mouse brain atlas (Paxinos and Franklin, 2001).

Regarding the pharmacological knowledge of antidepressants, the choice of KO mice as experimental models of anxiety–depression was remarkably appropriate because it is now well recognized that major depressive disorders result from a combination of genetic and environmental factors. In addition, knowing that anxiety and depression have a high co-morbidity (Gorman and Coplan, 1996; Leonardo and Hen, 2006), it is critical for basic research to develop animal models that present behavioral, neurochemical, and brain morphological phenotypes reminiscent of depression and anxiety. Some “serotonergic” KO mice display important changes in their basal phenotype. For example, constitutive 5-HT_1A_ receptor KO mice were simultaneously described by three different laboratories as an animal model of anxiety-related disorder (Heisler et al., 1998; Parks et al., 1998; Ramboz et al., 1998). They display decreased exploratory activity and increased fear of aversive environments and exhibited a decreased immobility in the FST, an effect commonly associated with antidepressant treatment. Brain microdialysis performed in 5-HT_1A_ receptor KO mice have proven to be a valuable technique to address key questions regarding the mechanism of action of antidepressants. One of the most interesting applications of microdialysis is to allow the study of basal extracellular levels of neurotransmitters, for example, in 5-HT_1A_ receptor KO mice. While conventional microdialysis does not allow reliable measurements of these basal levels (see Conventional Intracerebral *In Vivo* Microdialysis) the no net flux (or zero net flux) method of quantitative microdialysis in mutants allows the direct and accurate determination of basal extracellular levels of neurotransmitters (see Zero Net Flux Method of Quantitative* Intracerebral Microdialysis) The DRN is a brain region where 5-HText is known to regulate serotonergic transmission through activation of 5-HT_1A_ autoreceptors. When microdialysis was performed in the DRN, it was found that baseline DRN 5-HText did not differ between WT control and KO mice. This result suggests a lack of tonic control of 5-HT_1A_ autoreceptors on DR 5-HT release (Bortolozzi et al., 2004; Guilloux et al., 2006).

Furthermore, microdialysis helped to decipher the brain region-dependent effects of antidepressants. Both a saline injection and handling for 3 min increased DRN 5-HText in 5-HT_1A_ receptor KO mice, but not in control mice. Fluoxetine, a serotonergic antidepressant, induced a dose-dependent increase in DRN 5-HText in both genotypes, but this effect was markedly more pronounced in 5-HT_1A_ KO mice. These results suggest that the increased responsiveness of dialysate 5-HText in the DRN of 5-HT_1A_ receptor KO mice at least in part explain the anxious phenotype of these mutants. Such information can help to define a better treatment of anxiety-related disorders.

The inhibitory 5-HT_1A_ receptor exists in two separate populations with distinct effects on serotonergic signaling, i.e., an autoreceptor that limits 5-HT release throughout the brain and a heteroreceptor that mediates inhibitory responses to release 5-HT. Traditional pharmacologic and Tg strategies have tried to separate the distinct roles of these two receptor populations. Recently, Richardson-Jones et al. (2010) developed a new strategy to manipulate pre-synaptic 5-HT_1A_ autoreceptors in serotonergic raphe neurons without affecting 5-HT_1A_ heteroreceptors, generating mice with higher (1A-High) or lower (1A-Low) autoreceptor levels. In this latter line, it was thus possible to examine the brain 5-HT system by partially turning off 5-HT_1A_ autoreceptors at a specific time point and to study correlations between changes in 5-HT transmission and antidepressant-like activity of SSRIs in various behavioral tests. This strategy robustly affects raphe firing rates, but has no effect on either basal extracellular 5-HT levels as measured by *in vivo* microdialysis in the frontal cortex and ventral hippocampus. Interestingly, following 8 days of fluoxetine treatment, a difference in 5-HT levels was found in the hippocampus, with higher levels in the 1A-Low mice. In addition, 1A-Low mice displayed a larger increase in 5-HT in response to an acute challenge of fluoxetine in both brain regions. Together with electrophysiology data showing an increased spontaneous neuronal activity in the dorsal raphe of 1A-Low mice under stressful conditions, the microdialysis results were consistent with an increased serotonergic tone in these animals in response to an SSRI. Compared to 1A-Low mice, 1A-High mice show a blunted physiological response to acute stress, increased behavioral despair, and no behavioral response to antidepressant, thus modeling what we can find in patients with the 5-HT_1A_ risk allele. Indeed, human studies implicate a polymorphism in the promoter of the 5-HT_1A_ receptor gene in increased susceptibility to depression and decreased treatment response (Lemonde et al., 2003). These mice may thus, be conceived as a human equivalent to SSRI response (1A-Low) and resistance (1A-High; Blier, 2010). These results establish a causal relationship between 5-HT_1A_ autoreceptor levels and response to antidepressants.

The same group of researchers used a recently developed genetic mouse system to independently manipulate 5-HT_1A_ autoreceptor and heteroreceptor populations. They found that 5-HT_1A_ autoreceptors affect anxiety-like behavior, while 5-HT_1A_ heteroreceptors affect responses to forced swim stress, without effects on anxiety-like behavior (Richardson-Jones et al., 2011). These results establish distinct roles for the two receptors’ populations, providing evidence that signaling through endogenous 5-HT_1A_ autoreceptors is necessary and sufficient for the establishment of normal anxiety-like behavior.

Taken together, these data obtained in KO mice brought a lot of information about the pathophysiology of psychiatric disorders and their treatments.

Thus, in 2012, we have at our disposal a large number of genetically engineered mice, some of them being interesting animal models of anxiety and depression. These mice are very helpful to discover the underlying pathological mechanisms that limit the effects of current treatments of major depressive episodes and to identify the nature of the molecular cascades leading to the installation of disorders such as anxiety and depression. In addition, KO mice help to study the effects of acute and chronic treatment with antidepressants.

Recent advances in experimental approaches using genetically manipulated mice have already been summarized in the literature (Sotnikova and Gainetdinov, 2007). Knowing the large number of KO mice generated to date, it is not possible to detail the findings of each putative model interesting in the anxiety and depression field of research (SERT^-^^/^^-^ mice, Bengel et al., 1998; NK1 receptor KO mice, Froger et al., 2001; Guiard et al., 2004; β-arrestin 2 KO mice, Beaulieu et al., 2008). Therefore, the remainder of the present chapter will only describe some examples, which explain these statements.

## ADVANTAGES AND LIMITATIONS OF USING MICRODIALYSIS IN KO MICE

Depressive disorders result from a combination of genetic and environmental factors. To date, several genes appear to have in humans and animals, a greater influence than the other and emerge from the literature. Among them, the presence of a polymorphism of either SERT (Bengel et al., 1998; Kuzelova et al., 2010), 5-HT_1A_ receptor (Lemonde et al., 2003), the tryptophan hydroxylase type 2 (TPH-2; Invernizzi, 2007), or BDNF (Chen et al., 2006) is associated with the occurrence of depression related to stress, or to a response to behavioral tests predictive of the antidepressant-like activity of a molecule (Porsolt et al., 1977; Steru et al., 1985).

### ADVANTAGES

In these KO mice, we can measure, for example, the paradigms of stress to predict the antidepressant potential of a molecule and the selectivity of behavioral responses in comparison with non-mutated control animals: if these responses are diminished or absent in KO mice deprived of a gene encoding a neurotransmitter receptor, we may conclude that this receptor plays a major part either in the antidepressant-like effect and/or of the molecule. Regarding microdialysis, changes in dialysate levels of neurotransmitters following acute (Malagié et al., 2001) or chronic (Gardier et al., 2003) SSRI treatment can highlight the mechanism of action of these drugs.

Thus, we combined KO mice and receptor antagonist strategies to investigate the contribution of the 5-HT_1B_ receptor subtype in mediating the effects of an SSRI, paroxetine in mice (Malagié et al., 2001). Using microdialysis, we found that a single systemic administration of paroxetine (1 or 5 mg/kg by the i.p. route) increased 5-HText in the ventral hippocampus and frontal cortex of WT control and mutant mice. However, in the ventral hippocampus, the SSRI induced a larger increase in dialysate 5-HT levels in KO 5-HT_1B_ mice than in control mice. In addition, either the absence of the 5-HT_1B_ receptor (in KO 5-HT_1B_ mice) or its pharmacological blockade with the mixed 5-HT_1B_/1D receptor antagonist, GR 127935 (in WT mice) potentiated the effect of a single administration of paroxetine on extracellular 5-HT levels in the ventral hippocampus. Thus, these data underline several points:

(a) complementary results were obtained by combining KO mice and receptor antagonist strategies.
(b) there were already *in vitro* studies showing the role of terminal 5-HT_1B_ autoreceptors *in vivo* to control 5-HT release and reuptake (in slices; Pineyro et al., 1995). Our microdialysis data in KO 5-HT_1B_ mice brought additional information by suggesting that 5-HT_1B_ autoreceptors limit the effects of SSRIs on dialysate 5-HT levels at serotonergic nerve terminals and revealed the importance of a particular brain region, the ventral hippocampus. It is interesting to notice that recently, many experimental arguments have accumulated to suggest that antidepressants exert their behavioral activity in adult rodents, at least in part, by inducing of cellular and molecular changes in the adult hippocampus (David et al., 2010).
  By using microdialysis, we can also study changes in dialysate 5-HT levels in the DRN (see Introduction). Data described above in 5-HT_1A_ receptor KO mice illustrated this important contribution. This experiment can give further information when combined with measurements of the electrical activity of 5-HT neurons. Again, the comparison of results between a KO mice model and WT mice is very informative.

Neurochemical changes as measured by using microdialysis can have functional consequences since they correlated with behavioral data obtained, for example, in the FST. Three examples can illustrate these benefits.

Example 1, in WT mice: intra-hippocampal BDNF infusion can potentiate paroxetine-induced increase in 5-HText in the hippocampus (**Figure 2A**). The antidepressant-like activity of paroxetine as measured on swimming behavior was potentiated by BDNF (**Figure 2B**). These data suggest an interesting synergy between BDNF and SSRI on 5-HT neurotransmission; thus, such a co-administration improved the antidepressant-like activity of the SSRI (Deltheil et al., 2008, 2009).

Example 2, in 5-HT_1A_ receptor KO mice: as described in Guilloux et al. (2006), paroxetine (1 and 4 mg/kg) dose-dependently increased cortical 5-HText in both WT and KO genotypes, but the effects were greater in mutants (**Figure 3A**). Paroxetine administration also dose-dependently decreased the immobility time in both strains of mice, but the response was much greater in 5HT_1A_^-^^/^^-^ mice (**Figure 3B**). Overall these results suggest that the genetic inactivation of 5-HT_1A_ receptors, abolished the inhibitory feedback control exerted by somatodendritic 5-HT_1A_ autoreceptors, thus enhancing the response of mutant mice to stressful conditions such as the FST. Thus, following SSRI administration, an indirect activation of pre-synaptic 5-HT_1A_ receptors by endogenous 5-HT may limit its antidepressant-like effects in the FST in WT mice.

**FIGURE 4:**
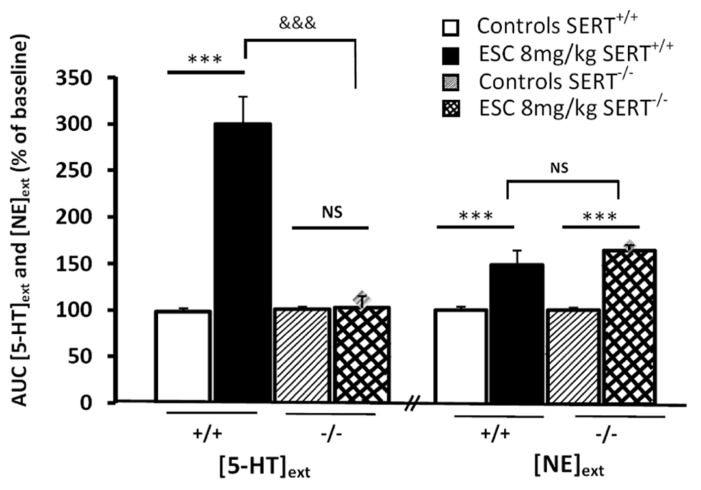
**Effect of systemic administration of escitalopram (ESC) on extracellular levels of 5-HT and noradrenaline (NE) in the frontal cortex inWT (SERT^+^^/^^+^) and KO (SERT^–/–^) mice.** AUC values (means ± SEM) were calculated for the amount of 5-HT and NE outflows collected during the 0–120 min post-treatment period (from Nguyen et al., 2013). ***p < 0.001 significantly different between controls and escitalopram-treated mice. ^&&&^*p* < 0.001 significantly different from SERT^-^^/^^-^ mice. NS, not statistically significant.

Example 3, in SERT^-^^/^^-^ mice: another interest of brain microdialysis is to allow the measurement of several neurotransmitters in the same sample. Thus, we recently examined the effects of the S(+)-enantiomer of citalopram, escitalopram (ESC) on both [5-HT]ext and extracellular levels of [NE]ext in the frontal cortex (FCx) of freely moving WT and mutant mice lacking SERT^-^^/^^-^ by using ICM (Nguyen et al., 2013). In WT mice, a single systemic administration of escitalopram produced a significant increase in cortical [5-HT]ext and [NE]ext (**Figure 4**). As expected, escitalopram failed to increase cortical [5-HT]ext in SERT^-^^/^^-^ mice, whereas its neurochemical effects on [NE]ext persisted in these mutants. In addition, in WT mice submitted to the FST, escitalopram increased swimming parameter without affecting climbing behavior (Nguyen et al., 2013).

### LIMITATIONS

There are also limits regarding the use of constitutive KO mice. Compensatory events may occur when mice are generated by homologous recombination (Gardier, 2009). For example, 5-HT_1B_ receptor KO mice exhibit a higher efficacy of 8-OH-DPAT-induced hypothermia suggesting that an adaptive thermoregulatory process involving the functional activity of somatodendritic 5-HT_1A_ receptors is altered in 5-HT_1B_ receptor KO mice (Gardier et al., 2001). By contrast, Bouwknecht et al. (2002) found no indications for adaptive changes in pre-synaptic 5-HT_1A_ receptor function in 5-HT_1B_ receptor KO mice as measured telemetrically on body temperature and heart rate responses.

Indeed, to study the direct consequences of alterations in the targeted gene, constitutive KO mice are very valuable tools because of compensatory processes that have taken place in reaction to life-long changes in gene expression (Groenink et al., 2003). The constitutive deletion of the NET, for example, induced an up-regulation of two other monoamine transporters DAT and SERT (Solich et al., 2011). An increase in the binding of [^3^H]paroxetine to the SERT and [^3^H]GBR-12935 to the DAT was observed in various brain regions of NET-KO mice, without alterations of mRNA encoding these transporters, as measured by in situ hybridization. This important finding obviously impacts the interpretation of previous data. Similarly, in SERT^-^^/^^-^ mice, Zhou et al. (2002) reported that 5-HT was found in DA neurons of homozygous (-/-), but not of heterozygous (+/-) mutant mice. DA neurons containing 5-HT have been observed in the substantia nigra and ventral tegmental area (VTA), but not in other brain areas of SERT^-^^/^^-^ mice. To verify the role of the DA transporter in such ectopic uptake, SERT^-^^/^^-^ mice were treated with DA uptake blocker GBR-12935: ectopic 5-HT in DA neurons was disappeared. These data indicate that 5-HT can be taken into DA neurons in rodents when SERT is not functionally adequate to remove extracellular 5-HT levels, and (c) the DA transporter is responsible for the 5-HT uptake into DA neurons. Thus, cross neuronal type uptake exists and serves as a compensatory backup when a specific transporter is dysfunctional. Thus, when using mice lacking an important protein from the earliest period of their existence, one has to be aware that compensatory alterations may occur in the brain as well as at the periphery. This point must be considered when it comes to interpretation of the experimental results.

**Table 1** summarizes the main advantages as well as some critical points of the intracerebral microdialysis technique.

## CONCLUSION

These past 25 years, different strains of KO mice became extremely valuable tools in Neuropharmacology. They help to identify in animals susceptibility genes and proteins involved in the pathological processes leading to anxiety and depression. These biological markers could then be helpful to pose the diagnosis of the disease in human. They also give information on their functional role, thus offering opportunities to develop new drug treatments. When performed in KO mice, and together with other techniques, brain microdialysis was very useful to define central monoaminergic dysfunctions having behavioral consequences similar to those associated with endogenous depression in humans. Some KO mice with mutations of serotonin targets (e.g., the 5-HT transporter SERT, 5-HT_1B_, 5-HT_1A_, and 5-HT4 receptors) display changes in phenotypes similar to those induced by chronic treatment with antidepressants in WT control mice.

Chronic antidepressant treatment may regulate the expression of neurotrophic factors such as BDNF and stimulate the process of adult neurogenesis in the dentate gyrus of the hippocampus in rats (Malberg et al., 2000) and adult mice (Santarelli et al., 2003; David et al., 2009). Changes in adult neurogenesis are only seen after chronic, but not acute, antidepressant treatment. Microdialysis studies in heterozygous mice for BDNF (Szapacs et al., 2004; Deltheil et al., 2008, 2009; Guiard et al., 2008) contributed to this knowledge by exploring the relationship between the hippocampal 5-HT system (i.e., the function of its transporter, one of the main targets of antidepressants) and brain BDNF levels.

In the future, our efforts to understand the pathophysiology of mood disorders, especially anxiety/depression, will focus on the antidepressant responses, especially in non-stressed and stressed rodents. Microdialysis technique in young or adult KO mice will continue to decipher region-dependent relationships between brain neurotransmitters and circuits involved in the mechanism of action of an antidepressant drugs’ polytherapy, soon available on the market. Furthermore, original strategies are now available to rescue the expression of a particular receptor subtype in a tissue-specific and temporally controlled manner in mice. For example, it is well known that agonists of the 5-HT_1A_ receptor such as buspirone have anxiolytic properties, and KO mice lacking this receptor show increased anxiety-like behavior (as indicated above). However, the relevant brain regions involved in anxious phenotype have not been delineated. Using such a tissue-specific, conditional rescue strategy for the 5-HT_1A_ receptor, Gross et al. (2002) engineered mice in which the expression of the 5-HT_1A_ receptor gene was under the control of the antibiotic doxycycline. The gene of interest was switched off when the mice were fed with the antibiotic. They used autoradiography to demonstrate that high levels of post-synaptic 5-HT_1A_ receptor expression in the hippocampus and cortex of the rescue mice, but the pre-synaptic 5-HT_1A_ autoreceptor, was undetectable in the raphe nuclei. By using mice in which the 5-HT_1A_ receptor can be knocked out at will, they show that the absence of the receptor in newborns lead to anxiety-like behavior, whereas its knock-out during adult life has no effect. In addition, they found that postnatal developmental processes help to establish adult anxiety-like behavior. Generating such a rescue mice is a long-lasting process, but each animal can be used as its own control.

Another strategy can be used to rescue a gene of interest, in which the KO mice line previously generated was used as the control group. A gene of interest is re-expressed into the midbrain of KO mice by stereotaxically injecting a lentiviral vector carrying this gene coding for a receptor to test for the selectivity of behavioral effects. This strategy was recently applied to study the role of beta2-subunit of the nicotinic acetylcholine receptor (nAChR; Maskos et al., 2005) in mediating the reinforcement properties of nicotine. In this example, microdialysis experiments were performed to confirm the rescue of nicotine effects in the vectorized line of mice compared to WT and KO lines. Regarding the serotonin field of research, global disruption of 5-HT2A receptor signaling in mice reduces inhibition in conflict anxiety paradigms without affecting depression-related behaviors. Selective rescue of 5-HT2A receptor in the cortex normalized conflict anxiety behaviors (Weisstaub et al., 2006). These findings indicate a specific role for cortical 5-HT2A receptors in the modulation of anxiety. These techniques allow greater precision and flexibility to generate KO rodents for understanding neurotransmitter function. No doubt that such novel and powerful tools, together with techniques of knock-in or SiRNA recently applied to the field of 5-HT receptors, will continue to give unexpected information on molecular and cellular mechanisms involved in mood disorders and their treatments.

## Conflict of Interest Statement

H. Lundbeck A/S, supported the preclinical study with escitalopram.
